# Supplementary material for: Oligodendroglia vulnerability in the human dorsal striatum in Parkinson’s disease
Source: Acta Neuropathol. 2025 May 5;149(1):46. doi: 10.1007/s00401-025-02884-5 (PMC12053221; doi:10.1007/s00401-025-02884-5)
Supplement: Supplementary file 1 — Supplementary file1 (DOCX 50858 KB) [file 401_2025_2884_MOESM1_ESM.docx]

# Supplementary Data

Supplementary Figure 1: Quality control metrics for the snRNA-seq dataset.

Supplementary Figure 2: Immunohistochemical Analysis of Tyrosine Hydroxylase, α-Synuclein, and MBP in CN Region.

Supplementary Figure 3: Cell Distribution Across Conditions and Regions.

Supplementary Figure 4: Subpopulation Percentages by Sex and Region.

Supplementary Figure 5: Hotspot Modules and Marker Genes Associated.

Supplementary Figure 6: Slingshot Pseudotime Analysis of Differentiation Trajectories Across Subpopulations.


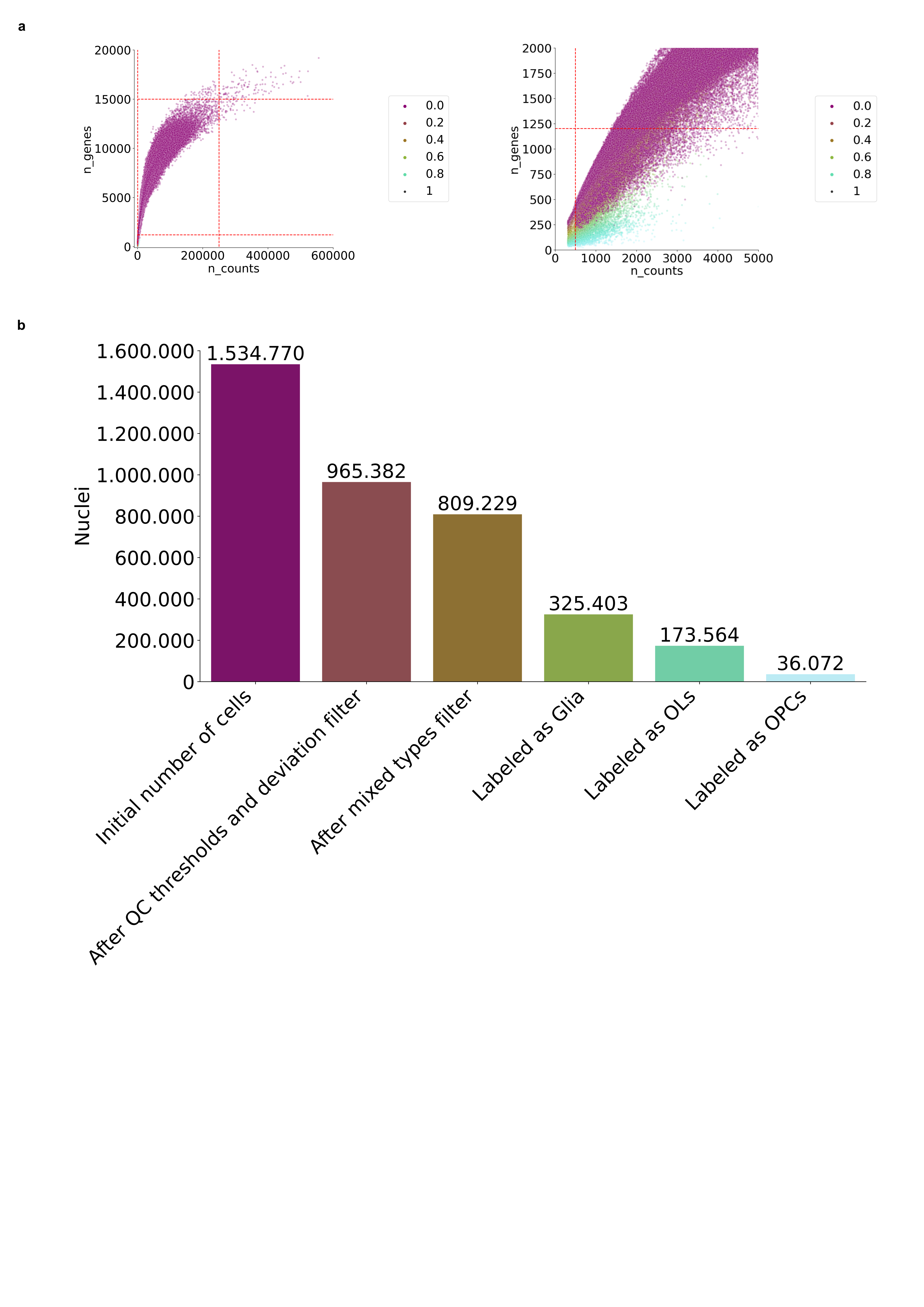


**Supplementary Figure 1.** Quality control metrics for the snRNA-seq dataset. Scatter plots with the distribution of gene counts and total counts per nucleus, stratified by the proportion of mitochondrial gene-derived UMIs among the total detected UMIs. Red lines indicate the values selected for the initial thresholding criteria (refer to Methods for details) (**a**). Summary of the number of nuclei retained at each stage of the analytical workflow (**b**).


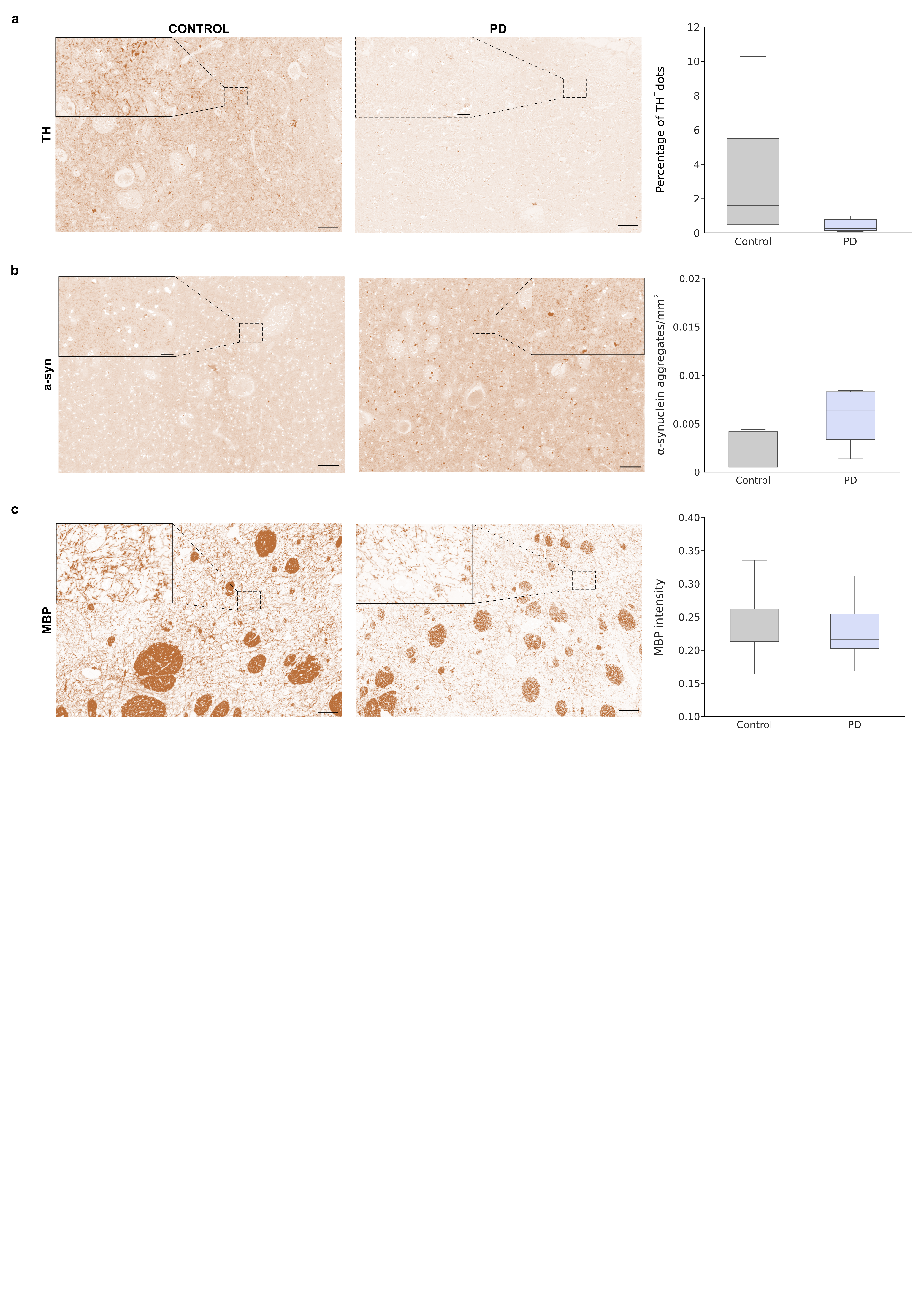


**Supplementary Figure 2.** Immunohistochemical Analysis of Tyrosine Hydroxylase, α-Synuclein, and MBP in CN Region. Immunohistochemical analysis of Tyrosine Hydroxylase in the CN region. Violin plots illustrate the distribution of marker expression in Control and PD conditions (**a**).Immunohistochemical analysis of α-synuclein in the CN region. Violin plots illustrate the distribution of marker expression in Control and PD conditions (**b**). Immunohistochemical analysis of MBP in the CN region. Violin plots illustrate the distribution of marker expression in Control and PD conditions. Statistical significance was determined using t-tests, with p-values indicated (< 0.05) (**c**).





**Supplementary Figure 3.** Cell Distribution Across Conditions and Regions.UMAPs display the uniform distribution of cells from each condition across all subpopulations (Control – Left, PD – Right) (**a**). UMAPs display the uniform distribution of cells from each region across all subpopulations (Caudate – Left, Putamen – Right) (**b**).


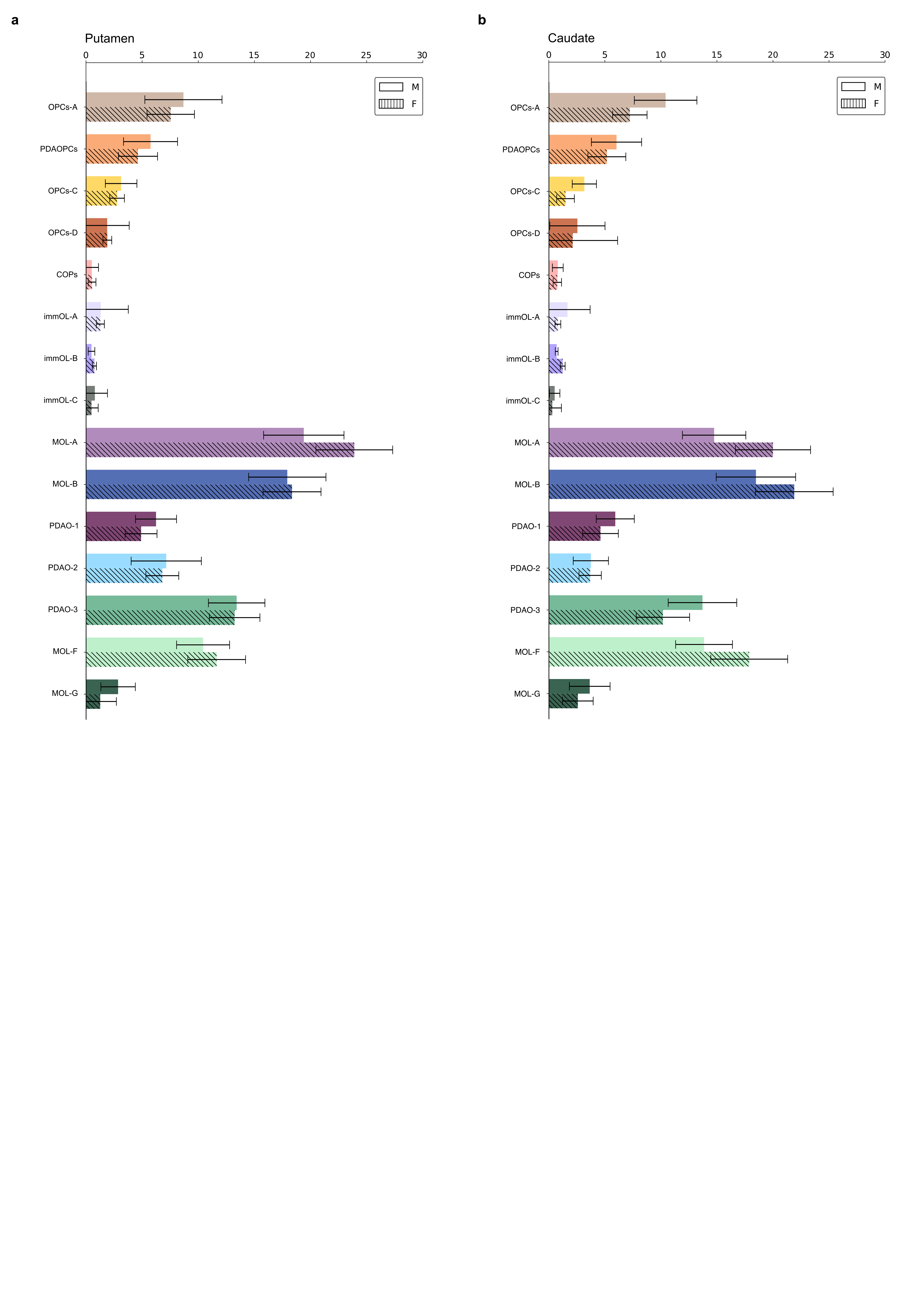


**Supplementary Figure 4.** Subpopulation Percentages by Sex and Region. Barplots showing the percentages of each subpopulation, calculated based on the total number of nuclei depending on the sex and Pu area (**a**). Barplots displaying percentages of each subpopulation, calculated based on the total number of nuclei depending on the sex and CN region (**b**).


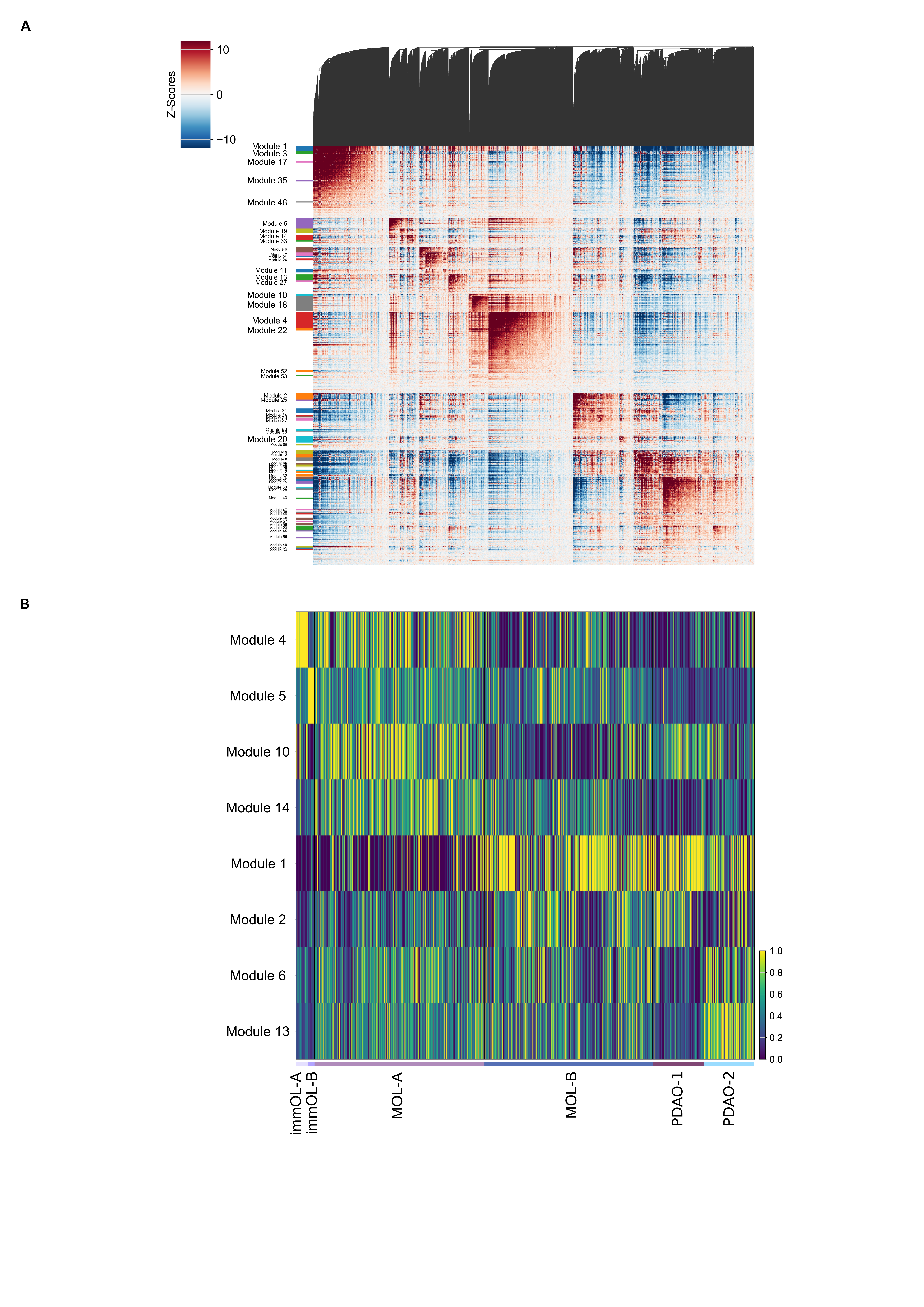


**Suplementary Figure 5.** Hotspot Modules and Marker Genes Associated.Heatmap illustrates modules detected by Hotspot analysis, based on local correlations within the dataset (**a**). Heatmap representing the association of modules with specific marker genes for each subpopulation, highlighting the distinct genetic signatures of the identified subpopulations (**b**).


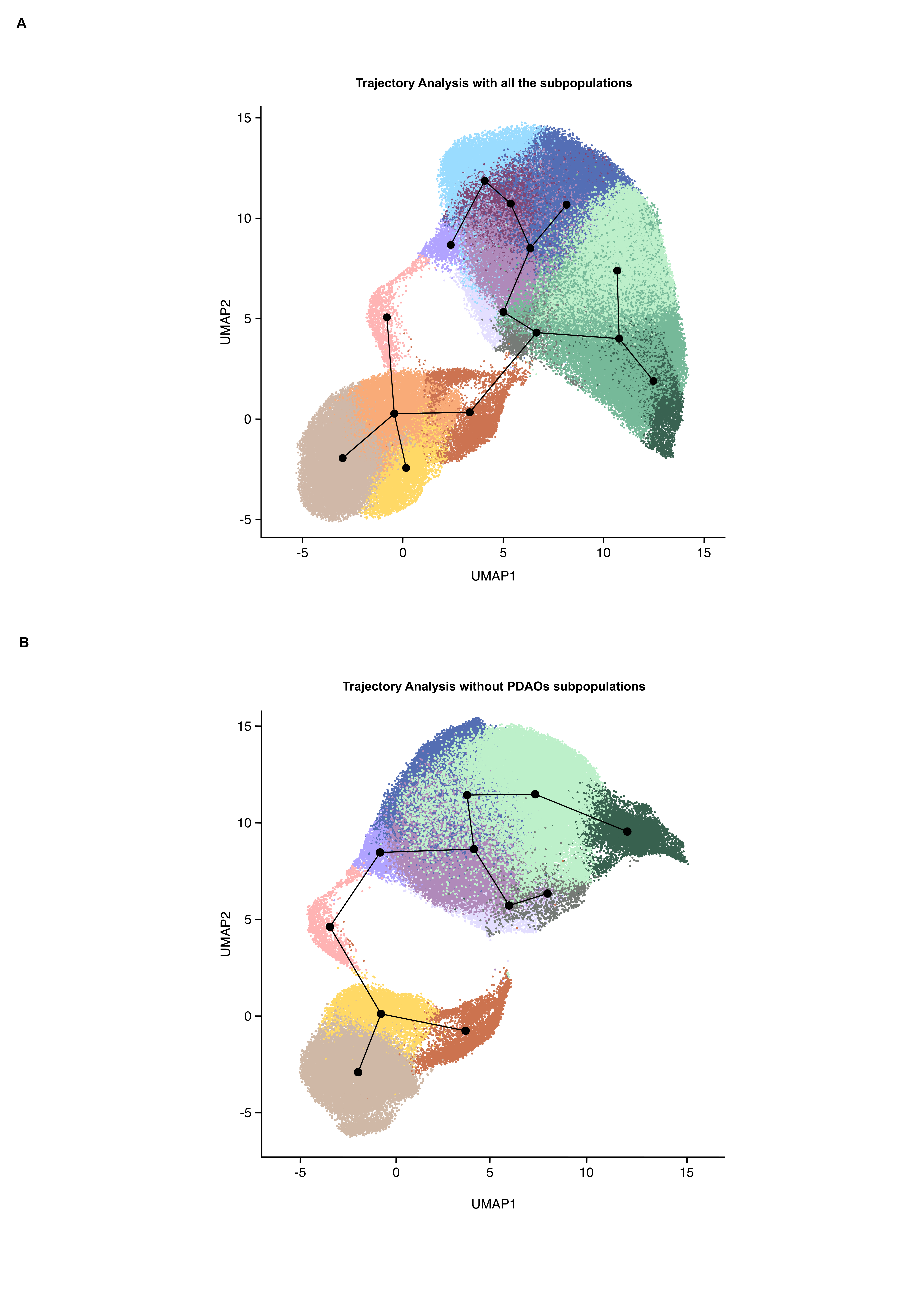


**Supplementary Figure 6.** Slingshot Pseudotime Analysis of Differentiation Trajectories Across Subpopulations. Pseudotime UMAP generated via Slingshot including all the subpopulations. Each dot is related to a specific subpopulation, while lines indicate how the differentiation process occurs (**a**). UMAP visualization of differentiation trajectory excluding PDA clusters (**b**).
